# Ophthalmologic school-based screening revealing Kearns-Sayre syndrome: a case report

**DOI:** 10.11604/pamj.2022.41.226.33085

**Published:** 2022-03-18

**Authors:** Amine Ennejjar, Salma Moutamani, Taha Boutaj, Wiame Touil, Abdellah Amazouzi, Ouafa Cherkaoui

**Affiliations:** 1 Ophthalmology Department “A”, Ibn Sina University Hospital *(Hôpital des Spécialités)*, Mohammed V University, Rabat, Morocco,; 2 Neurology Department “B”, Ibn Sina University Hospital *(Hôpital des Spécialités)*, Mohammed V University, Rabat, Morocco

**Keywords:** Kearns-Sayre syndrome, pigmentary retinopathy, ptosis, atrioventricular block, case report

## Abstract

Kearns-Sayre syndrome is a rare mitochondrial disorder. It had a triad of features, including progressive external ophthalmoplegia, pigmentary retinopathy, and an alteration of cardiac conduction. The ocular manifestations include bilateral ptosis, progressive external ophthalmoplegia, and atypical pigmentary retinopathy. We report the case of a 9-year-old Moroccan patient who has been diagnosed with Kearns-Sayre syndrome during an ophthalmologic school-based screening. This case highlights the interest of school-based screening in the diagnosis and management of a rare disease.

## Introduction

Kearns-Sayre Syndrome (KSS) is a rare mitochondrial cytopathy. It was first described in 1958 [1]. It had a triad of features: progressive external ophthalmoplegia, pigmentary retinopathy, and an alteration of cardiac conduction [2]. Other signs can be seen in the frame KSS such as myopathy, hyperproteinorachia, cerebellar ataxia, nephropathy, dental abnormalities, hypoparathyroidism, hypogonadism, diabetes insipidus, deficiency in growth hormone, and maculopathy [3]. We report the case of a 9-year-old Moroccan who has diagnosed Kearns-Sayre syndrome during an ophthalmologic school-based screening.

## Patient and observation

**Patient information:** a 9-year-old Moroccan male, the second of 3 children with 2^nd^ degree of consanguineous parents, came in a standard medical screening in a primary school. He was presented with 3 years past medical history of bilateral ptosis and lipothymic discomfort.

**Figure 1:**
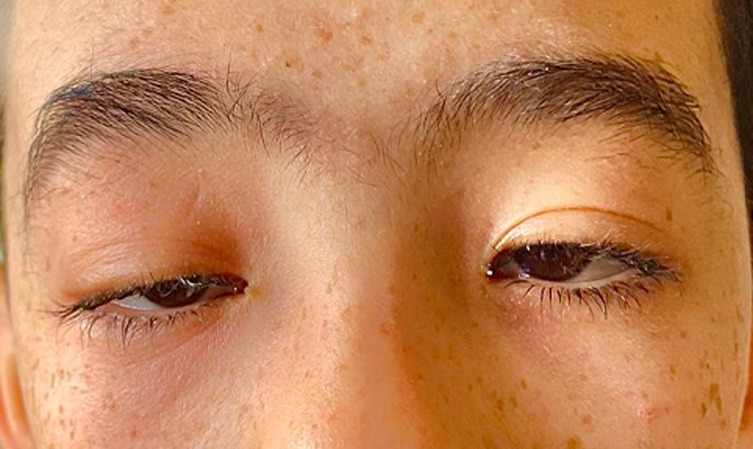
photography of the patient showing bilateral ptosis

**Figure 2:**
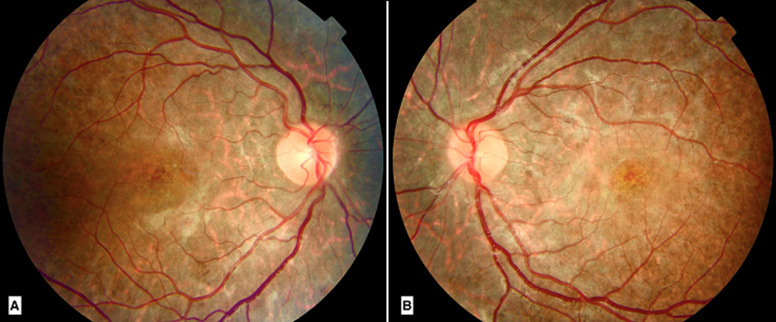
funduscopy of the right eye (A); and the left eye (B); bilateral pigmentary retinopathy with macular dystrophy

**Clinical findings:** general examination found short stature with a BMI within normal limits (17kg/m^2^). On the first examination, the visual acuity was 20/100 in the right eye and 20/80 in the left eye. The inspection found bilateral ptosis (Figure 1). Ocular motility examination revealed partial external ophthalmoplegia with mild limitations in gaze in all directions. The pupillary light reflex was present. Intraocular pressure was good in both eyes. Slit-lamp biomicroscopic examination found a calm anterior segment. After dilatation, funduscopy of both eyes showed bilateral atypical pigmentary retinopathy with macular dystrophy (Figure 2). The diagnosis of KSS was suspected. The patient was referred to the cardiology and neuropediatric department. Examination found normal cardiac auscultation and normal neurological assessment.

**Figure 3:**
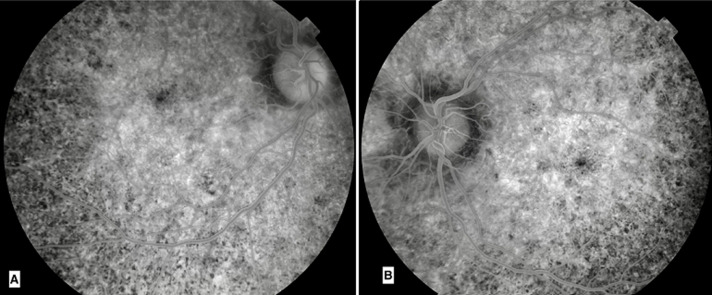
fluorescein angiography of the right eye (A); and the left eye (B); areas of hyper- and hypofluorescence “aspect of salt and pepper”

**Figure 4:**
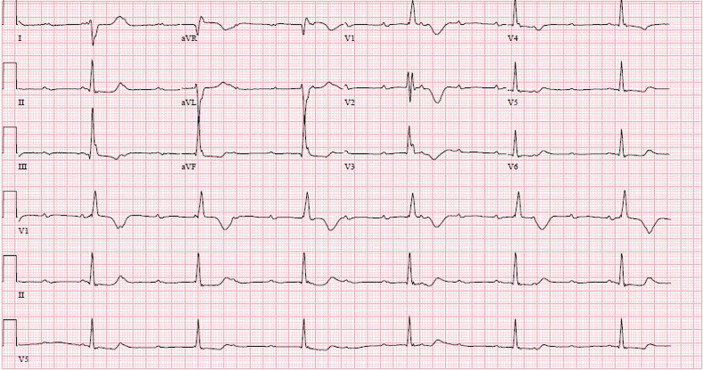
third degree atrioventricular block on 12-lead electrocardiogram

**Diagnostic assessment:** fluorescein angiography found areas of hyper- and hypofluorescence called “aspect of salt and pepper” (Figure 3). The electrocardiogram revealed a 3^rd^ degree atrioventricular block with an average ventricular rate at 37 b/min (Figure 4). The biological assessment including thyroid testing, hepatic balance, glycemia, lactic acid, renal function, blood cell, and urine analysis was normal, except for creatine phosphokinases (347 UI/L), and lactates (241 mg/L). A biopsy of the deltoid muscle found ragged red fibers with Gomori Trichome.

**Diagnosis:** the KSS diagnosis was retained following clinical triad: bilateral ptosis, pigmentary retinopathy, and an alteration of cardiac conduction, and then confirmed by muscular biopsy.

**Figure 5:**
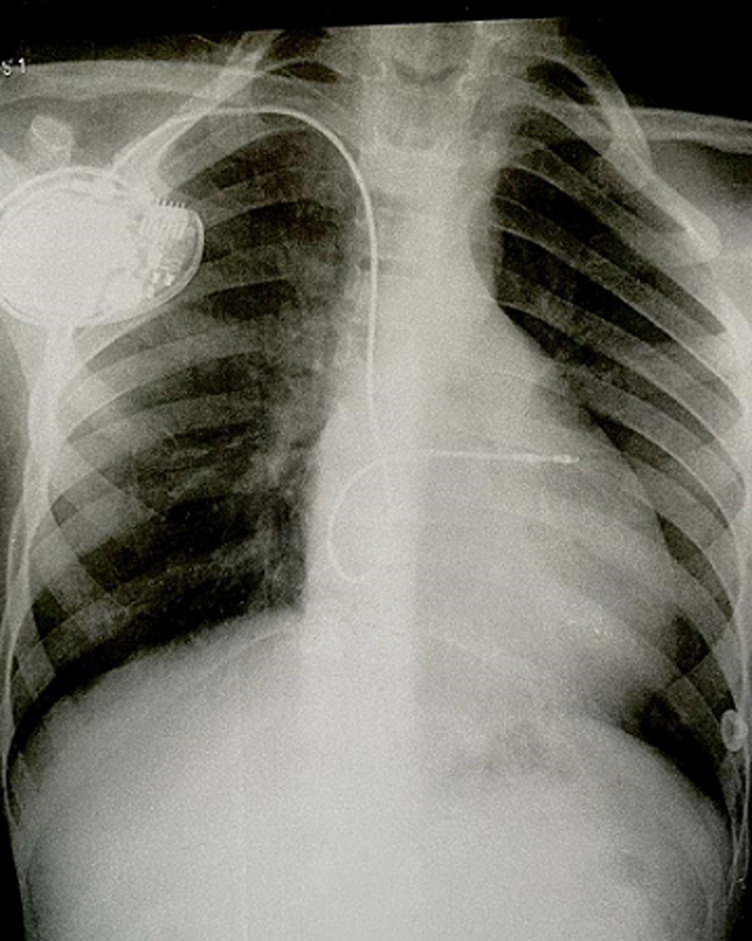
pacemaker implantation on chest X-ray

**Therapeutic interventions:** it was decided to realize the implantation of a double chamber pacemaker (Figure 5).

**Follow-up and outcome of interventions:** follow-up after one week was uneventful.

**Patient perspective:** the young boy was very satisfied during his hospitalization and after the treatment. The patient was delighted with the care he received and was optimistic about the outcome of his condition.

**Informed consent:** children under the age of 18 are not allowed to give consent. Informed consent was obtained from his parents for us to publish the case report and use the pictures.

## Discussion

Described in 1958 at Mayo Clinic by Thomas P. Kearns and George P. Sayre [1], KSS is a rare mitochondrial disorder. It is caused by rearrangements of the mitochondrial genome including various deletions and/or duplications ranging from 1000 to 10 000 DNA nucleotides. The most common deletion is 4.9 kb [4]. Most often are sporadic. However, in about 15% of cases, it may be autosomal dominant or recessive [3]. Shemesh A. and Margolin E. have reported a prevalence of 1.6 cases per 100,000 in the Finnish population [5]. The age of onset is generally below 20. It can affect striated muscles, the myocardium, the central or peripheral nervous system, the skin, and the retinal pigment epithelium [6].

The first and mainly common sign is bilateral ptosis because of the involvement of the levator muscle. The second sign is progressive external ophthalmoplegia (PEO): the pupil continues to react, but the eye muscles are paralyzed. Ptosis and PEO are the most frequent ophthalmologic signs in KSS [4]. Cardiac involvement is variable. Half of patients can develop conductive abnormalities leading to complete atrioventricular block or bradycardia induced polymorphic ventricular tachycardia [7]. Other disorders associated includes dementia, cerebellar ataxia, deafness, nystagmus, protein levels, exercise intolerance, proximal myopathy, thyroid disorders, Addison´s disease, hypoparathyroidism, dysphagia due to achalasia, and renal tubular acidosis [3]. The degree of vision loss depends on retinal damage, in particular macular. The main differential diagnosis of KSS are myasthenia gravis, other mitochondrial disorders (mitochondrial myopathy with lactic acidosis (MELAS), maternally inherited diabetes and deafness syndrome, myoclonic epilepsy, and ragged-red fibers (MERRF) syndrome), orbital myositis, and thyroid disease [5].

In order to confirm the diagnosis of KSS, a muscle biopsy is realized and studied under light microscopy with Gomori staining, in which ragged red fibers are evident. There is currently no proven treatment for KSS [3,5]. The treatment is supportive. Some studies suggest coenzyme Q10. But it does not improve the ocular manifestations [8]. Surgery may be needed to correct eyelid weakness especially when the ptosis is important, but there is a risk of recurrence and possible ocular complications. Kearns-Sayre syndrome is associated with a high risk of sudden death following complete atrioventricular block [9]. A permanent pacemaker is recommended in high-grade heart blocks. It has been shown to improve the prognosis [6,9]. Surveillance includes yearly ECG, echocardiography, and 24-hour Holter monitoring (regardless of patient's age), audiometry, and endocrinologic evaluation [8].

## Conclusion

Kearns-Sayre syndrome is a rare disease entity with important clinical implications regarding ophthalmological manifestations, cardiac evaluation, and pacemaker implantation. There should be a clinical suspicion when a patient presents with the classic triad to initiate an early palliative treatment. Follow-up can prevent possible complications.
